# Using wearable technology to objectively investigate physical behaviour and determine health outcomes of a physical activity intervention in patients with psoriasis

**DOI:** 10.1002/ski2.473

**Published:** 2024-11-19

**Authors:** Rory Sheppard, Weh K. Gan, Gladys L. Onambele‐Pearson, Helen S. Young

**Affiliations:** ^1^ Division of Musculoskeletal and Dermatological Sciences School of Biological Sciences The University of Manchester Manchester UK; ^2^ The Dermatology Centre Salford Royal Hospital Manchester Academic Health Science Centre The University of Manchester Manchester UK; ^3^ Department of Sport and Exercise Sciences Manchester Metropolitan University Manchester UK

## Abstract

**Background:**

Sedentary behaviour has a detrimental effect on health independent of the amount of physical activity undertaken. Despite the association of cardiometabolic and psychosocial comorbidities with psoriasis, how physical behaviour influences health outcomes in patients with psoriasis is poorly understood.

**Objectives:**

We hypothesized that objective measurement of physical behaviour, using wearable digital technology, would have utility in understanding the clinical impact of an exercise intervention designed in partnership with patients with psoriasis.

**Methods:**

Fourteen patients with psoriasis completed a 20‐week study. During weeks 1–10, participants followed an incrementally progressive, exercise intervention, followed by independent activities during weeks 11–20. Accelerometers (GENEActiv Original) and pedometers recorded physical behaviour. Evaluation at week‐0, ‐10, and ‐20 included assessment of psoriasis, cardiometabolic disease/risk, psychological health and functional capacity.

**Results:**

Our intervention supported significantly increased physical activity, including moderate–vigorous physical activity (*p* = 0.04) and step count (*p* = 0.04). We also observed a significant association between physical activity and psoriasis area and severity index (PASI)‐50 response (*p* = 0.01) and psychosocial functioning (*p* = 0.029) together with a significant negative correlation between step count and psoriasis severity (*p* = 0.012). We observed no change in total waking hour sedentary behaviour.

**Conclusion:**

Objective measurement of physical behaviour, using wearable digital technologies, offers a mechanism to further understand the clinical impact of lifestyle behaviour interventions. Crucially, despite increased levels of physical activity, we observed no change in total waking hour sedentary time. Further investigation is required to establish how modification of physical behaviour could offer an adjuvant management strategy for patients with psoriasis.

**What is already known?**

- Sedentary behaviour has a detrimental effect on health and wellbeing independent of the amount of physical activity undertaken.
- A physical activity intervention which increases physical activity is associated with a significant improvement in psoriasis severity, cardiometabolic disease status, psychological health, and functional capacity.

**What does this study add?**

- Digital technology can be used to record patterns of physical behaviour in patients with psoriasis.
- Total accumulated waking hour sedentary behaviour remained unchanged following our physical activity intervention, though a significant improvement in moderate–vigorous physical activity was observed.
- We report a significant negative correlation between physical activity and psoriasis severity, suggesting that as physical activity increases a reduction in the severity of psoriasis is observed.
- Further investigation to establish whether modification of physical behaviour offers an adjuvant management strategy for patients with psoriasis is required.

## 1 INTRODUCTION

Psoriasis is a chronic inflammatory skin disease associated with an increased risk of developing cardiometabolic disease. Patients with psoriasis often fail to achieve the quantity of physical activity recommended for the promotion of health and wellbeing due to psoriasis‐specific barriers which limits participation.^1^ We previously reported that supporting increased physical activity to meet current AHA guidelines in patients with psoriasis was associated with improved health outcomes, including significantly improved psoriasis control, reduced cardiovascular disease risks, enhanced wellbeing and increased functional capacity.^2^

Sedentary behaviour, which describes the total waking time spent in a seated, reclined or lying position,^3^ has a detrimental effect on health and wellbeing independent of the amount of physical activity undertaken.^4^, ^5^ In recent years, engagement in prolonged sedentary behaviour has gained attention as a distinctive risk factor associated with chronic disease onset.^6^ Indeed, previous work in our group identified that patients with psoriasis aged 18–65 spend a median of 360‐min sitting per day,^7^ as assessed by the international physical activity questionnaire (IPAQ), which is 50% more time than a similar cohort of individuals without psoriasis.^8^ Importantly, it is only at supramaximal levels of moderate–vigorous activities (≥420‐min/week, ∼60‐min/day), that physical activity manages to offset the negative health effects of a high sedentary time, but such activity levels are not realistic for most.^4^, ^9^ However, breaking up sedentary time with short bouts of light‐intensity physical activity offers a potential solution,^10^ and we and others have observed health outcomes from this approach, including reduced cardiometabolic disease risk^11^ and all‐cause mortality.^12^

Investigators have traditionally relied on subjective instruments to evaluate physical activity/sedentary behaviour such as questionnaires and physical activity diaries.^13^, ^14^ These tools have many advantages, including cost‐effectiveness, simplicity to administer/collect data and their general acceptance by researchers and clinicians in the field.^15^ However, the administration of self‐reported tools has several limitations including recall and reporting bias^13^ and reliance on participant interpretation/understanding of questions. Additionally, these instruments were recently found to have moderate validity in reporting total physical activity and moderate‐to vigorous intensity physical activity, and poor validity was shown in reporting sedentary behaviour amongst a young adult population.^16^ Taken together, reliable and consistent methods are needed to overcome these limitations,^14^ which may be obtained through the use of data‐driven technologies to objectively and accurately quantify physical behaviour.^14^ Indeed, NHS England advocates digital technology as a means to improve healthcare delivery, patient experience and clinical outcomes.^17^, ^18^ To that end, we hypothesized that it is essential to objectively measure both the amount/intensity of physical activity and the duration of sedentary time when assessing the overall physical behaviour profile of an individual and that this would have utility in understanding the clinical impact of our physical activity intervention, designed with and for patients with psoriasis.

## 2 MATERIALS AND METHODS

### 2.1 Study population

Patients (*n* = 19) aged 18–60 years, with measurable but clinically stable moderate‐to‐severe chronic plaque psoriasis, with/without stable psoriatic arthritis were recruited to the study.

### 2.2 Study design

**FIGURE 1:**
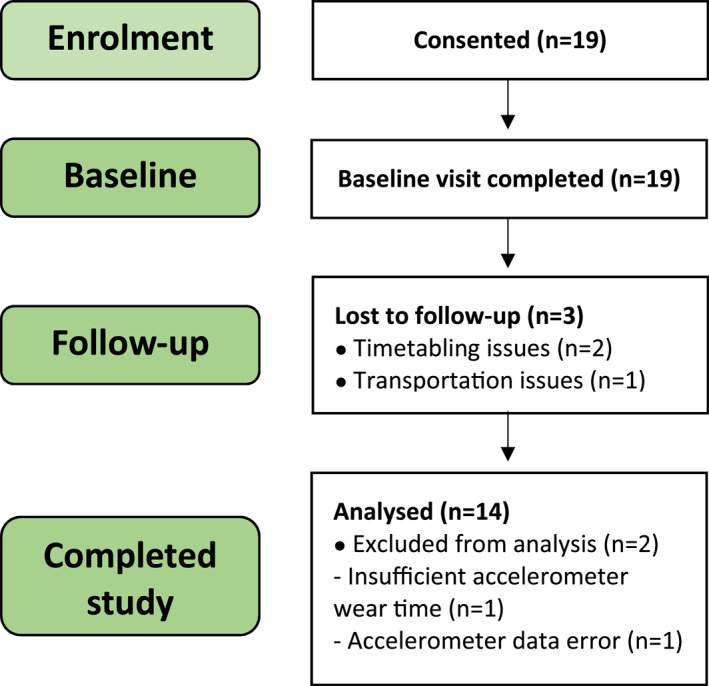
Recruitment, attrition and completion data of volunteers in the study.

Our 20‐week prospective cohort study was approved by the Local Research Ethics Committee (20/NW/0443) and conducted in accordance with the Declaration of Helsinki principles. Written informed consent was obtained prior to study participation. During the first 10‐weeks, participants completed a physical activity intervention comprising twice‐weekly walking sessions, each lasting one‐hour and led by a sport and exercise scientist, as previously described.^19^ Participants achieved health‐promoting levels of exercise within 4‐weeks of participation and each session incorporated separate pre‐exercise stretching, warm‐ups and cool‐downs. In the second part of the study (weeks 11–20) participants followed independent activities (Figure 1). Clinical assessment was completed at baseline and at the week‐10 and ‐20 time points.

### 2.3 Measurement of physical behaviour using wearable devices

A hip‐worn pedometer (Onwalk 900; Decathlon Group, Villeneuve d’Ascq, France) was used by participants throughout the study (weeks 0–20) to record their daily step count and details of independent activities into a physical activity diary. A wrist‐worn accelerometer (GENEActiv Original; Activinsights Ltd, Kimbolton, UK), which is a lightweight and waterproof device that records raw accelerations in three axes (x,y,z) into datafiles, with sufficient memory to store up to 60 days of physical behaviour data,^20^ was used during the first 10‐weeks of this study. The GENEActiv device has been validated in our previous studies,^21^ and was selected for this study following workshops with lived‐experience contributors to co‐design our physical activity intervention, as previously described.^19^

Participants wore the accelerometers continuously, even whilst washing and sleeping. Accelerometer data was recorded at a frequency of 60 Hz, to capture sufficient data points over the collection period in line with previous studies of adult populations.^22^, ^23^ The data was uploaded to a computer and the bin files were converted to 10‐s epoch.csv files using GENEActiv PC software version 3.3. The accelerometer data was imported into a custom‐built Microsoft Excel spreadsheet that calculated daily time spent in sedentary behaviours (sitting, inactive standing), physical activity (moderate‐vigorous and light‐intensity physical activity), and non‐activity time at night (a surrogate measure of sleep).

### 2.4 Clinical assessment of participants at baseline, mid‐intervention, and on completion of study

Clinical assessments of psoriasis were made using psoriasis area and severity index (PASI; 0–72),^24^ dermatology life quality index (DLQI; 0–30 points)^25^ and physicians global assessment (PGA; 0–7 points). Blood pressure was measured and validated tools were used to assess psychological health (36‐Item Short Form Health Survey; SF‐36) and functional capacity.

### 2.5 Statistical analysis

All analyses were performed using SPSS statistical software (version 28.0.1.0; Armonk, NY, USA). Sample size calculations suggested 15 participants were needed to detect changes in the primary and secondary outcome measures, DLQI and blood pressure, at a statistical power of 80% and alpha level set at 0.05. This was based on a five‐point change in DLQI assuming a standard deviation (SD) of 5.14,^26^ and 5mmHg reduction in systolic blood pressure with a SD 6.32mmHg.^27^ To allow for a 20% dropout rate we aimed to recruit 18 participants. Normality of data was tested using the Shapiro–Wilk test. Comparisons between accelerometer variables were made using a paired *t*‐test, or Wilcoxon signed ranks test for non‐parametric datasets. Intergroup comparisons were made using the Mann–Whitney *U* test. Missing accelerometer data was imputed using the last observation carried forward statistical approach. All other variables were compared using a one‐way analysis of variance with repeated‐measures test or Friedman test followed by post‐hoc Wilcoxon signed ranks test for non‐parametric datasets. Spearman correlations were used to determine relationships between timepoint differences in step count and PASI, DLQI, and PGA. Descriptive statistics were presented as median and inter‐quartile range (IQR) for non‐parametric datasets. Significance level was set at *p* < 0.05.

## 3 RESULTS

The final cohort consisted of 14 patients (8 men and 6 women; Figure 1), with a median age of 39.0 (IQR: 30.8–46.8), median BMI of 27.1 (IQR: 25.1–31.9) and of whom 21.4% had psoriatic arthritis (Table 1).

**TABLE 1:** Baseline characteristics of the patients with psoriasis.

Characteristic	All participants (*n* = 14)	Men (*n* = 8)	Women (*n* = 6)
Age and anthropometric measurements			
Age, yr	39.0 (30.8–46.8)	38.0 (29.3–48.0)	39.0 (34.0–42.5)
Height, cm	1.67 (1.63–1.76)	1.76 (1.69–1.81)	1.64 (1.60–1.65)
Body weight, kg	81.7 (71.7–88.5)	79.9 (76.8–89.5)	85.1 (68.4–86.9)
Body mass index, kg/m^2^	27.1 (25.1–31.9)	26.1 (24.1–28.9)	30.8 (26.5–31.9)
History of psoriasis			
Age of onset, yr	19.0 (15.3–20.8)	20.5 (18.3–24.0)	14.0 (13.0–18.0)
Disease duration, yr	19.5 (11.3–28.8)	12.5 (9.5–28.3)	21.0 (19.3–28.0)
Family history of psoriasis, *n* (%)	7 (50.0%)	4 (50.0%)	3 (50.0%)
Nail involvement, *n* (%)	5 (35.7%)	4 (50.0%)	1 (16.7%)
Current psoriasis treatment, *n* (%)			
Topical	12 (85.7%)	7 (87.5%)	5 (83.3%)
Phototherapy	0 (0.0%)	0 (0.0%)	0 (0.0%)
Systemic	1 (7.1%)	0 (0.0%)	1 (16.7%)
Biological	2 (14.3%)	2 (25.0%)	0 (0.0%)
Previous psoriasis treatment, *n* (%)			
Topical	13 (92.9%)	7 (87.5%)	6 (100.0%)
Phototherapy	3 (21.4%)	2 (25.0%)	1 (16.7%)
Systemic	3 (21.4%)	2 (25.0%)	1 (16.7%)
Biological	2 (14.3%)	2 (25.0%)	0 (0.0%)
Past medical history, *n* (%)			
Psoriatic arthritis	3 (21.4%)	1 (12.5%)	2 (33.3%)
Cardiovascular disease	1 (7.1%)	1 (12.5%)	0 (0.0%)
Chronic kidney disease	0 (0.0%)	0 (0.0%)	0 (0.0%)
Hypertension	5 (35.7%)	3 (37.5%)	3 (33.3%)
Angina	2 (14.3%)	2 (25.0%)	0 (0.0%)
Rheumatoid arthritis	0 (0.0%)	0 (0.0%)	0 (0.0%)
Diabetes mellitus	1 (7.1%)	1 (12.5%)	0 (0.0%)
Depression	3 (21.4%)	2 (25.0%)	1 (16.7%)
Asthma	2 (14.3%)	1 (12.5%)	1 (16.7%)

*Note*: The data are presented as median and interquartile range for continuous variables, and percentages for categorical variables, *n* = 14.

Abbreviations: *n*, number of participants; yr, years.

The wear‐time for inclusion in our analysis was 8–18 h/day, based on previous literature which demonstrated that a minimum of 8 h wear‐time is required to reliably estimate activity during waking hours using accelerometry,^28^ and a maximum of 18 h wear would prevent misclassification of behaviour whilst sleeping.^29^ Sleeping intervals were not included in the analysis.

Engagement in sedentary behaviours were classified as either (i) daily waking time spent in a sitting or reclining posture with an energy expenditure equivalent to ≤1.5 metabolic equivalents (METs)^6^ or ii) inactive standing, defined as daily time spent in a standing posture with limited movement eliciting ≤1.5 METs.^30^ Engagement in physical activity was classified as either moderate–vigorous physical activity, which represents daily time spent in standing activity eliciting ≥3 metabolic equivalents (METs),^31^ or light‐intensity physical activity, defined as daily time spent in a standing activity eliciting 1.5–2.9 METs.^31^

### 3.1 Physical activity was significantly improved at week 10

Following our intervention, at week‐10, we observed a significant improvement in moderate–vigorous physical activity compared to baseline, although light‐intensity physical activity did not achieve statistical significance (week‐0: median 0.2 [IQR: 0.2–0.3] h; week‐10: median 0.3 [IQR: 0.2–0.3] h, *p* = 0.491; Table 2). Compared to baseline, moderate–vigorous physical activity significantly increased by 0.5 h/day at week‐10 (week‐0: median 1.0 [IQR: 0.7–1.7] h/day; week‐10: median 1.5 [IQR: 1.0–2.0] h/day, *p* = 0.042); a 1.9% improvement in daily time spent engaged in moderate–vigorous physical activity (week‐0: median 4.3% of day [IQR: 2.9–7.1]; week‐10: median 6.2% of day [IQR: 4.3–8.4], *p* = 0.038; Table 2).

**TABLE 2:** Assessments of sedentarism, physical activity, and a surrogate measure of sleeping time at baseline (week‐0) and the primary study endpoint (week‐10).

Characteristic	Baseline (Week 0)	Mid‐intervention (Week 10)	*p* value
			Week 0–10
Assessments of sedentarism			
Sedentary behaviour (h/day)	12.3 (11.5–13.7)	12.3 (11.7–13.8)	0.491
Sedentary behaviour (% of day)	51.1 (47.7–57.0)	51.2 (48.6–57.4)	0.482
Standing time (h/day)	0.3 (0.2–0.7)	0.4 (0.2–0.5)	0.831
Standing time (% of day)	1.4 (1.0–2.9)	1.5 (0.9–1.9)	0.833
Assessments of physical activity			
Moderate‐vigorous PA (h/day)	1.0 (0.7–1.7)	1.5 (1.0–2.0)	0.042
Moderate‐vigorous PA (% of day)	4.3 (2.9–7.1)	6.2 (4.3–8.4)	0.038
Light‐intensity PA (h/day)	0.2 (0.2–0.3)	0.3 (0.2–0.3)	1.000
Light‐intensity PA (% of day)	1.0 (0.7–1.4)	1.1 (0.7–1.4)	1.000
Surrogate assessment of sleep			
Sleeping time (h/day)	9.2 (9.1–10.1)	9.1 (8.7–9.4)	0.049
Sleeping time (% of day)	38.4 (37.9–42.2)	37.8 (36.1–39.2)	0.043

*Note*: The data are presented as median and interquartile range for continuous variables, and percentages for categorical variables, *n* = 14.

Abbreviation: PA, physical activity.

During the final week of the physical activity intervention, participants accumulated ≥2016 MET‐min/week of total activity per person. This indicates that participants completed physical activity independent to and in addition to those delivered by the physical activity intervention. Furthermore, the total volume of physical activity exceeded the minimum activity recommendations 4‐fold.^32^ Other measures of physical activity revealed step count was significantly increased at week‐10 (7791 [IQR: 4084–9716]; *p* = 0.004), compared to baseline (4596 [IQR: 3127–6776]; Table 2).

### 3.2 Measures of sedentarism remained unchanged at the week 10 endpoint

We observed no significant changes in measures of sedentarism following our intervention and during the first 10‐weeks of our study, as assessed by total sedentary time per day (week‐0: median 12.3 [IQR: 11.5–13.7] h/day; week‐10: median 12.3 [IQR: 11.7–13.8] h/day, *p* = 0.491), percentage of day spent sedentary (week‐0: median 51.1% of day [IQR: 47.7–57.0]; week‐10: median 51.2% of day [IQR: 48.6–57.4], *p* = 0.482), daily standing time (week‐0: median 0.3 [IQR: 0.2–0.7] h; week‐10: median 0.4 [IQR: 0.2–0.5] h, *p* = 0.831 and percentage of day in standing time (week‐0: median 1.4% of day [IQR: 1.0–2.9]; week‐10: median 1.5% of day [IQR: 0.9–1.9], *p* = 0.833; Table 2). We also observed a significant increase in non‐activity time at night at week‐10 (week‐0: median 9.2 [IQR: 9.1–10.1] h/day; week‐10: median 9.1 [IQR: 8.7–9.4] h/day, *p* = 0.049) and percentage of daily non‐activity time at night (week‐0: median 38.4% of day [IQR: 37.9–42.2]; week‐10: median 37.8% of day [IQR: 36.1–39.2], *p* = 0.038), which was used as a surrogate for sleep (Table 2).

Sub‐group analysis revealed that compared with baseline, sedentary behaviour in women (*n* = 6) was reduced by 78 min/day at week‐10 (week‐0: median 13.6 [IQR: 12.8–13.7] h/day; week‐10: median 12.3 [IQR: 11.7–13.2] h/day, *p* = 0.491), which amounted to a 5.3% reduction in daily time spent engaged in sedentary behaviour (week‐0: median 56.7% of day [IQR: 53.2–57.0]; week‐10: median 51.4% of day [IQR: 48.8–55.1], *p* = 0.491), though statistical significance was not achieved.

### 3.3 Increased physical activity is negatively correlated with psoriasis severity and is associated with PASI‐50 response

Spearman correlation analysis revealed a moderate but statistically significant negative correlation between step count and PASI over the first 10‐weeks of the study (*r* = −0.57, *n* = 14, *p* = 0.012, one‐tailed), suggesting that the increased step count was accompanied by a significant reduction in PASI. We also observed a moderate but significantly negative correlation between step count and PGA during the first 10‐weeks of the study (*r* = −0.53, *n* = 14, *p* = 0.025, one‐tailed), suggesting that as step count increased, PGA reduced. However, we found no relationship between physical behaviour and clinically meaningful improvement in blood pressure, SF‐36 PCS and MCS, or functional capacity.

We then investigated whether physical behaviour was associated with clinical outcomes, defining clinically meaningful improvement as: (i) PASI‐50, (ii) 5‐point reduction in DLQI from baseline or achieving a DLQI of 1 or 0 from a baseline of ≥3, (iii) a 5mmHg reduction in blood pressure, (iv) 5‐point reduction in SF‐36 physical component summary (PCS) and mental component summary (MCS), (v) 2 additional repetitions in the 30‐s sit‐to‐stand, (vi) 0.9‐s reduction in timed up‐and‐go and (vii) 24.1‐s increase in single leg balance for functional capacity. Those achieving a defined clinically meaningful improvement were designated ‘responders’ and those not achieving these outcomes were designated ‘non‐responders’.

We observed a statistically significant difference in median step count between baseline and week‐20 when comparing participants achieving a meaningful reduction in DLQI at week‐20 from those who did not (*p* = 0.029; Table 3). These data suggested that patients achieving a meaningful reduction in DLQI were more physically active than those who did not.

**TABLE 3:** Comparisons between responders and non‐responders of those achieving a clinically meaningful reduction in PASI, diastolic blood pressure, and DLQI, for measures of physical activity, sedentary behaviour, and energy expenditure at baseline (week‐0), the primary endpoint (week‐10), and secondary endpoint (week‐20).

Group	Variables compared	Responders	Non‐responders	Week	*p* value
PASI‐50	Step count	8967 (6659–9101)^a^	4264 (2720–6524)	0	0.085
PASI‐50	Step count	12,376 (7820–14456)^a^	6549 (4252–8508)	10	0.010
PASI‐50	Step count	6283 (4172–9571)^a^	4136 (3362–4436)	20	0.125
PASI‐50	Light‐intensity PA	0.3 (0.2–0.3)^a^	0.3 (0.2–0.4)	0	0.500
PASI‐50	Light‐intensity PA	0.3 (0.2–0.3)^a^	1.1 (0.8–1.2)	10	0.005
PASI‐50	Moderate‐vigorous PA	1.7 (0.9–1.8)^a^	0.3 (0.2–0.3)	0	0.101
PASI‐50	Moderate‐vigorous PA	2.3 (1.2–2.4)^a^	1.3 (1.1–1.7)	10	0.358
PASI‐50	Sedentary behaviour	12.5 (12.0–13.3)^a^	11.9 (11.1–13.6)	0	0.245
PASI‐50	Sedentary behaviour	11.7 (11.7–14.0)^a^	12.6 (11.7–13.9)	10	0.432
PASI‐50	Standing time	0.3 (0.3–0.3)^a^	0.4 (0.3–0.8)	0	0.177
PASI‐50	Standing time	0.4 (0.4–0.6)^a^	0.3 (0.2–0.4)	10	0.136
PASI‐50	MET‐minutes	333 (194–342)	72.0 (54.0–90.0)	0	0.106
PASI‐50	MET‐minutes	441 (242–459)^a^	342 (252–450)	10	0.418
Diastolic BP	Step count	5393 (4308–6524)^b^	3335 (2720–9101)	0	0.369
Diastolic BP	Step count	6439 (4123–10108)^b^	7820 (7156–8508)	10	0.298
Diastolic BP	Step count	6860 (3671–9813)^b^	4188 (3384–5316)	20	0.331
Diastolic BP	Light‐intensity PA	0.2 (0.2–0.3)^b^	0.3 (0.2–0.4)	0	0.144
Diastolic BP	Light‐intensity PA	0.7 (0.6–1.0)^b^	1.0 (0.5–1.2)	10	0.412
Diastolic BP	Moderate‐vigorous PA	0.2 (0.2–0.4)^b^	0.3 (0.2–0.4)	0	0.315
Diastolic BP	Moderate‐vigorous PA	1.2 (1.1–1.6)^b^	1.8 (0.9–2.0)	10	0.411
Diastolic BP	Sedentary behaviour	12.0 (11.2–13.3)^b^	12.0 (11.6–13.6)	0	0.397
Diastolic BP	Sedentary behaviour	12.8 (11.9–14.1)^b^	11.8 (11.6–13.4)	10	0.413
Diastolic BP	Standing time	0.3 (0.2–0.7)^b^	0.4 (0.3–0.5)	0	0.412
Diastolic BP	Standing time	0.3 (0.2–0.4)^b^	0.4 (0.3–0.5)	10	0.285
Diastolic BP	MET‐minutes	68 (56–92)^b^	90 (63–104)	0	0.308
Diastolic BP	MET‐minutes	338 (272–356)^b^	446 (279–450)	10	0.333
DLQI	Step count	4264 (2423–4308)^c^	5572 (3127–6776)	0	0.143
DLQI	Step count	1950 (1645–5519)^c^	4511 (3615–5961)	20	0.241
DLQI	Light‐intensity PA	0.3 (0.3–0.3)^c^	0.3 (0.2–0.4)	0	0.430
DLQI	Moderate‐vigorous PA	1.0 (0.6–1.0)^c^	0.2 (0.2–0.3)	0	0.240
DLQI	Sedentary behaviour	11.8 (11.5–12.1)^c^	12.0 (11.4–13.6)	0	0.345
DLQI	Standing time	0.8 (0.6–1.0)^c^	0.3 (0.3–0.5)	0	0.236
DLQI	MET‐minutes	203 (137–268)^c^	63 (54–90)	0	0.145

*Note*: The data are presented as median and interquartile range for continuous variables.

Abbreviations: BP, blood pressure; DLQI, Dermatology Life Quality Index; MET, Metabolic Equivalent of Task; PA, Physical Activity; PASI, Psoriasis Area and Severity Index.

^a^ PASI‐50 responders represent patients that achieved a 50% reduction in PASI.

^b^ Diastolic BP responders represents patients that achieved a 5mmHg reduction in diastolic BP.

^c^ DLQI responders represent patients that achieved a clinically meaningful reduction in DLQI (based on a 5‐point change in DLQI, or patients with a DLQI of 1 or 0 at week 20, in those with baseline DLQI of ≥3). *N* = 14.

At week‐10, significant differences in step counts were observed between PASI‐50 responders (median: 12376 [IQR: 10098–13416]) and non‐responders (median: 6549 [IQR: 4252–8508], *p* = 0.01; Table 3), and although step count was higher amongst responders at week‐20 (median: 6283 [IQR: 4172–9571]), compared to non‐responders (median: 4136 [IQR: 3362–4436], *p* = 0.125), statistical significance was not achieved (Table 3). We observed a difference in MET‐mins at week‐10 between PASI‐50 responders (median: 441 [IQR: 242–459]) and non‐responders (median: 342 [IQR: 252–450], *p* = 0.418), statistical significance was not achieved (Table 3). We also found that those achieving a PASI‐50 response at week‐10 engaged in less sedentary behaviour (median: 11.7 [IQR: 11.7–14.0] h/day), compared to those who did not achieve this benchmark (median: 12.6 [IQR: 11.7–13.9] h/day, *p* = 0.432), however, statistical significance was not achieved (Table 3).

## 4 DISCUSSION

This study was the first to examine patterns of physical behaviour and investigate whether these could influence health outcomes in patients with psoriasis. Adherence to our physical activity intervention, co‐designed with patients with psoriasis, resulted in significantly increased physical activity, including objective measurement of moderate–vigorous physical activity and step count. We evidenced an association between physical activity and clinical outcomes, including psoriasis control, PASI‐50 response and psychosocial functioning. In addition, we described a significant negative correlation between step count and psoriasis severity as measured by PASI and PGA, suggesting that as levels of physical activity increased, severity of psoriasis decreased. However, we observed no change in total accumulated waking hour sedentary time, as individuals continued to spend prolonged periods of time sitting or lying down, despite their increased levels of physical activity.

The use of self‐reported instruments such as IPAQ and global physical activity questionnaire as a measure of sedentary behaviour, compared to accelerometry, remains controversial. Although self‐reported instruments are useful for gathering physical activity data in large cohorts, these tools have low validity and reliability for measurement of sedentary behaviour.^33^ Our use of wearable digital technologies facilitated objective measurement of physical behaviour and we observed significant improvements in physical activity and step counts following our intervention. Levels of physical activity peaked at week‐10, following completion of the physical activity intervention, during which each session was led by a sport and exercise scientist, before returning to baseline levels of activity during weeks 11–20 (when participants followed independent activities rather than those led by our sports and exercise scientist). These findings are in line with other studies and suggest that lifestyle behaviour change can be initiated and maintained over short periods of time.^34^

Promisingly, we found that those achieving PASI‐50 had a significantly higher step counts than non‐responders, suggesting that increased physical activity may be associated with improved psoriasis control. Indeed, the anti‐inflammatory effects of exercise in the prevention and treatment of chronic diseases is well established^35^ with others reporting that a 10‐week vigorous‐intensity walking intervention in patients with stable rheumatoid arthritis was associated with reduced disease activity, including improved innate immune function and cardiorespiratory fitness.^36^

Crucially, we observed no significant changes in total waking hour sedentarism following our intervention suggesting that patients may continue to have an increased risk of adverse health outcomes, despite significant improvement in the volume of physical activity undertaken.^3^ Although, we found no significant relationship between sedentary behaviour and psoriasis, cardiometabolic and psychological health outcomes, this was likely due to unchanged (and high) levels of sedentarism throughout the study. It is increasingly clear that reducing overall sedentary time, interrupting bouts of prolonged (≥30‐min) sedentary behaviour whilst increasing physical activity is associated with improved health outcomes and it is critically important to address this in future studies.^37^

This study had limitations. First, participant recruitment may have been influenced by self‐selection bias. However, the final cohort, comprised individuals having a range of psoriasis severities and comorbidities which were representative of the psoriasis population. Second, our study cohort was relatively small (after adjustment for dropouts, missing data, and errors), which may have limited the power of the study to detect changes. Nevertheless, the retention rate achieved in this study was significantly greater than those previously reported in other physical activity intervention studies.^38^ Third, our study had a single experimental arm and therefore in the absence of a control group it was not possible to determine whether our physical activity intervention could constitute a promising therapeutic intervention for patients with psoriasis. However, our data suggests that lifestyle interventions, which promote increased physical activity whilst reducing and interrupting prolonged bouts of sedentary behaviour, merit further investigation in appropriately powered randomized controlled trials. Finally, confounding factors may have influenced study outcomes. However, we mitigated for these by using inclusion/exclusion criteria to ensure only those with stable, measurable disease, with no recent medication changes were eligible to participate. Furthermore, any seasonal effects were mitigated by completing the intervention throughout the year.

## 5 CONCLUSION

In conclusion, objective measurement of physical behaviour, using wearable digital technologies offers a mechanism to further understand the clinical impact of lifestyle behaviour interventions, designed with and for patients with psoriasis. It is possible that modification of physical behaviour could offer a low cost, adjuvant management strategy, which is potentially beneficial across several critical health outcomes for patients with psoriasis. Further investigation of physical behaviour strategies, which reduce and interrupt periods of sedentary behaviour (factors that are independently related to negative health outcomes) whilst increasing tolerable physical activity in patients with psoriasis, are required.

## CONFLICT OF INTEREST STATEMENT

The authors declare that they have no conﬂicts of interest.

## AUTHOR CONTRIBUTIONS

**Rory Sheppard**: Data curation (equal); formal analysis (equal); investigation (equal); methodology (equal); software (equal); writing ‐ original draft (equal). **Weh K. Gan**: Data curation (supporting); investigation (supporting); methodology (supporting); writing ‐ original draft (supporting); writing ‐ review & editing (supporting). **Gladys L. Onambele‐Pearson**: Conceptualization (supporting); data curation (supporting); formal analysis (supporting); investigation (supporting); methodology (supporting); supervision (supporting); writing ‐ review & editing (supporting). **Helen S. Young**: Conceptualization (lead); data curation (equal); formal analysis (equal); funding acquisition (lead); investigation (supporting); methodology (supporting); project administration (lead); resources (lead); software (lead); supervision (lead); visualization (lead); writing ‐ original draft (supporting); writing ‐ review & editing (lead)

## ETHICS STATEMENT

The study was approved by the Local Research Ethics Committee (20/NW/0443).

## PATIENT CONSENT

Not applicable.

## Data Availability

The data underlying this article are available in the article.
